# Multiphoton microscopy for label-free multicolor imaging of peripheral nerve

**DOI:** 10.1117/1.JBO.27.5.056501

**Published:** 2022-05-16

**Authors:** Lars Rishøj, Iván Coto Hernández, Siddharth Ramachandran, Nate Jowett

**Affiliations:** a Boston University, Department of Electrical and Computer Engineering, Boston, Massachusetts, United States; b Technical University of Denmark, DTU Fotonik, Kgs. Lyngby, Denmark; c Mass Eye and Ear and Harvard Medical School, Surgical Photonics and Engineering Laboratory, Boston, United States

**Keywords:** lasers, imaging, label-free, nonlinear optical microscopy, nerve fibers, myelinated

## Abstract

**Significance:**

Means for quantitation of myelinated fibers in peripheral nerve may guide diagnosis and clinical decision making in management of peripheral nerve disorders. Multiphoton microscopy techniques such as the third-harmonic generation enable label-free *in vivo* imaging of peripheral nerves.

**Aim:**

Develop a multiphoton microscope based on a custom high-power infrared fiber laser for label-free imaging of peripheral nerve.

**Approach:**

A cost-effective multiphoton microscope employing a single fiber laser source at 1300 nm was designed and used for stain-free multicolor imaging of murine and human peripheral nerve.

**Results:**

Second-harmonic generation signal from collagen centered about 650-nm delineated neural connective tissue, whereas third-harmonic general signal centered about 433-nm delineated myelin and other lipids. In sciatic nerve from transgenic reporter mice expressing yellow fluorescent protein within peripheral neurons, three-photon-excitation with emission peak at 527-nm delineated axoplasm. The signal obtained from unlabeled axially sectioned samples was adequate for segmentation of myelinated fibers using commercial image processing software. In unlabeled whole mount specimens, imaging depths over 100-μm were achieved.

**Conclusions:**

A multiphoton microscope powered by a fiber laser enables stain-free histomorphometry of mammalian peripheral nerve. The simplicity of the microscope design carries potential for clinical translation to inform decision making in peripheral nerve disorders.

## 1 Introduction

Nonlinear optical microscopy (NLOM) comprises multiphoton fluorescence and harmonic generation techniques^1^^–^^3^ that enable imaging of biologic tissue including peripheral nerve at depths far exceeding those achievable by single-photon excitation microscopy.^4^^,^^5^ Label-free NLOM approaches for imaging peripheral nerve include coherent Raman scattering (CARS)^6^ microscopy, second harmonic generation (SHG),^7^^,^^8^ third harmonic generation (THG) microscopy,^9^ or a combination of SHG and THG imaging.^10^ Here, a custom-built fiber laser based on soliton self-mode conversion (SSMC)^11^ is employed for multi-color and high-resolution NLOM^12^ of murine and human peripheral nerve.^13^^,^^14^ Collagen is a primary constituent of the peripheral nerve sheath and extracellular matrix encasing individual axons; dysregulation in collagen production impacts nerve regeneration.^15^^,^^16^ Myelin is a lipid-rich substance that envelops select central and peripheral nervous system axons, providing mechanical protection and electrical insulation for rapid and efficient propagation of action potentials. Dysregulation of myelin production is a hallmark of manifold neurological disease states.^17^ In this paper, peripheral nerve sheaths, myelin sheaths, and individual axons are imaged using a custom single excitation source within the second optical window (∼1300  nm).^18^ Collagen-dense nerve sheaths (endoneurium, perineurium, and epineurium) are resolved using an SHG signal centered at ∼650  nm, whereas lipid-dense myelin sheaths of individual myelinated axons are resolved using a THG signal centered at ∼433  nm. Additionally, excitation wavelengths around 1300-nm enable 3PE of popular green and yellow fluorescent proteins [green fluorescent protein (GFP) and yellow fluorescent protein (YFP)]^19^ and two-photon excitation of several red-fluorescent-dyes.^20^ Here, stain-free multicolor imaging of peripheral nerve from transgenic mice expressing YFP under control of a modified thy1 promoter is achieved with capture of YFP emission maximum at 527 nm delineating the axoplasm of individual axons, and SHG and THG signals delineate neural connective tissue and myelin sheaths, respectively.

## 2 Experimental Setup and Biological Samples

**Fig. 1:**
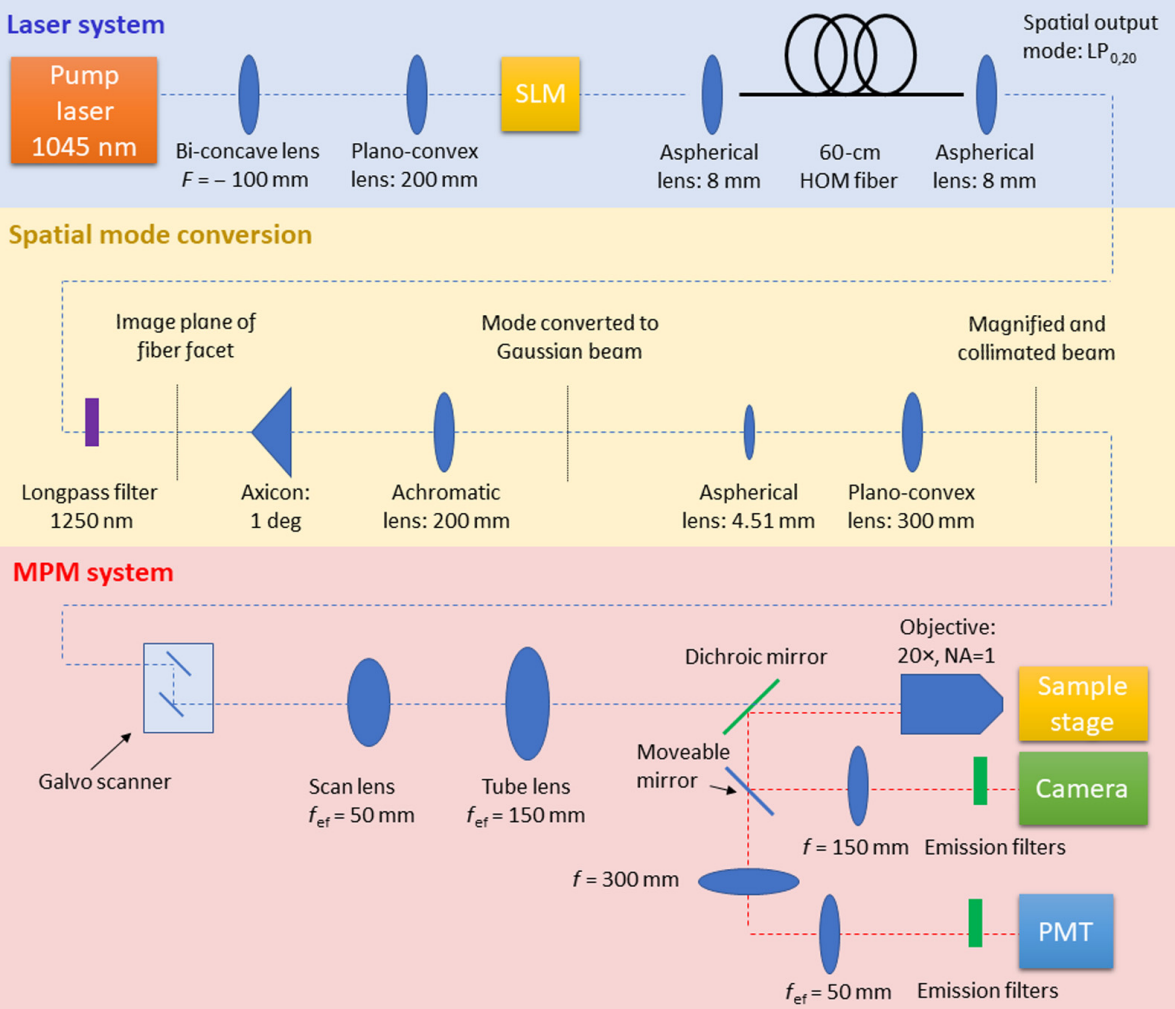
Experimental setup. The hardware employed comprises the laser system (blue shaded region), spatial mode conversion (yellow shaded region), and the MPM system (red shaded region).

A custom multiphoton microscope for multicolor imaging of peripheral nerve was designed and assembled. A custom fiber laser system based on SSMC with a tunable wavelength range from 1045 to 1320 nm was optimized at a single wavelength (∼1300  nm), selected to minimize biological sample damage while allowing for deep NLOM imaging. The experimental setup is shown in Fig. 1. It consists of three elements, the laser system (blue shaded region), spatial mode conversion (yellow shaded region), and the multiphoton microscopy (MPM) system (red shaded region), which are described below. More information about the laser system and spatial mode conversion can be found in Refs. 11 and 21.

### 2.1 Laser System

**Fig. 2:**
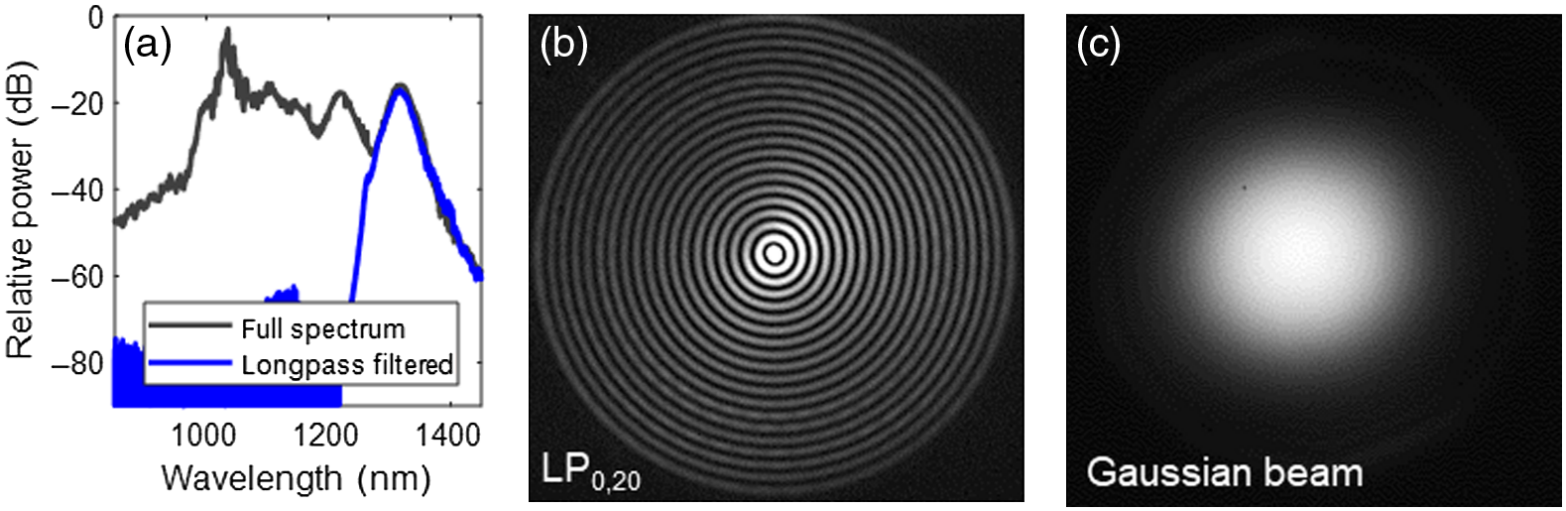
(a) Output spectrum before and after spectral filtering. (b) Experimental image of the ${\mathrm{LP}}_{0,20}$ output mode after spectral filtering. (c) Gaussian beam after spatial re-conversion.

The blue shaded region of Fig. 1 is the laser system, where a 100 fs pulse at 1045 nm from the pump laser (Y-Fi, KMLabs, Boulder, Colorado, United States) is converted to ∼1300  nm. A telescope magnifies the beam before propagation onto a spatial light modulator, which encodes a transverse spatial phase onto the incident Gaussian beam to ensure excitation of a single pure mode in the multimode fiber.^21^ Here, a linearly polarized (LP) ${\left(\mathrm{LP}\right)}_{0,21}$ mode is excited in the custom 54-cm higher-order-mode fiber (core diameter of 97  μm and numerical aperture of 0.34). Initially in the fiber, a soliton is formed at the pump wavelength in the ${\mathrm{LP}}_{0,21}$ mode and subsequently the center wavelength of the soliton continuously red-shifts via soliton self-frequency shifting (SSFS). The soliton eventually reaches a wavelength at which it has the same group velocity as the ${\mathrm{LP}}_{0,20}$ mode at a longer wavelength that is separated by a frequency detuning equal to the Raman gain peak of silica (13 THz). At this point, the energy of the soliton is transferred via the recently discovered nonlinear effect of SSMC to a new pulse in the ${\mathrm{LP}}_{0,20}$ mode. From this point, until the end of the fiber, the new pulse then frequency shifts via SSFS to 1317 nm where the pulse energy is measured to be 80 nJ.^11^^,^^22^ Figure 2(a) shows the output spectra from the fiber before and after spectral filtering with a 1250-nm longpass filter. Figure 2(b) shows the spatial mode of the pulse centered at 1317 nm. It is seen to be in the ${\mathrm{LP}}_{0,20}$ mode, which resembles a truncated Bessel beam comprising a central peak surrounded by 19 concentric rings. This exact frequency conversion process requires that light initially possessed certain linear properties, which is ensured by launching the light in a specific higher order mode in the multimode fiber, which in this case is the ${\mathrm{LP}}_{0,21}$ mode. Using an autocorrelator, the pulse duration was measured at 74 fs, corresponding to a peak power of ∼1.1  MW. Based on the measured spectral bandwidth, it was found that the pulses are nearly transform-limited, an expected result considering the pulses are solitons.

### 2.2 Spatial Mode Conversion

In the yellow shaded region of the experimental setup shown in Fig. 1, a longpass filter initially removes the residual pump light. Next, the ${\mathrm{LP}}_{0,20}$ fiber output mode is converted to a Gaussian-like beam using an axicon and lens, with a measured power conversion efficiency of 81% (theoretically 86%).^11^ Figure 2(c) shows the image of the Gaussian beam after the spatial mode conversion. The following two lenses magnify and collimate the Gaussian beam to a beam diameter of 5 mm [full width at half maximum (FWHM)]. At this point, the pulse duration was measured at 100 fs using an autocorrelator. Though Bessel beams may be used to extend the depth of focus and for fast volumetric imaging of sparse samples,^19^ a Gaussian beam was utilized as it is more energy-efficient for imaging histological sections of excised nerves.

### 2.3 Multiphoton Microscope

**Fig. 3:**
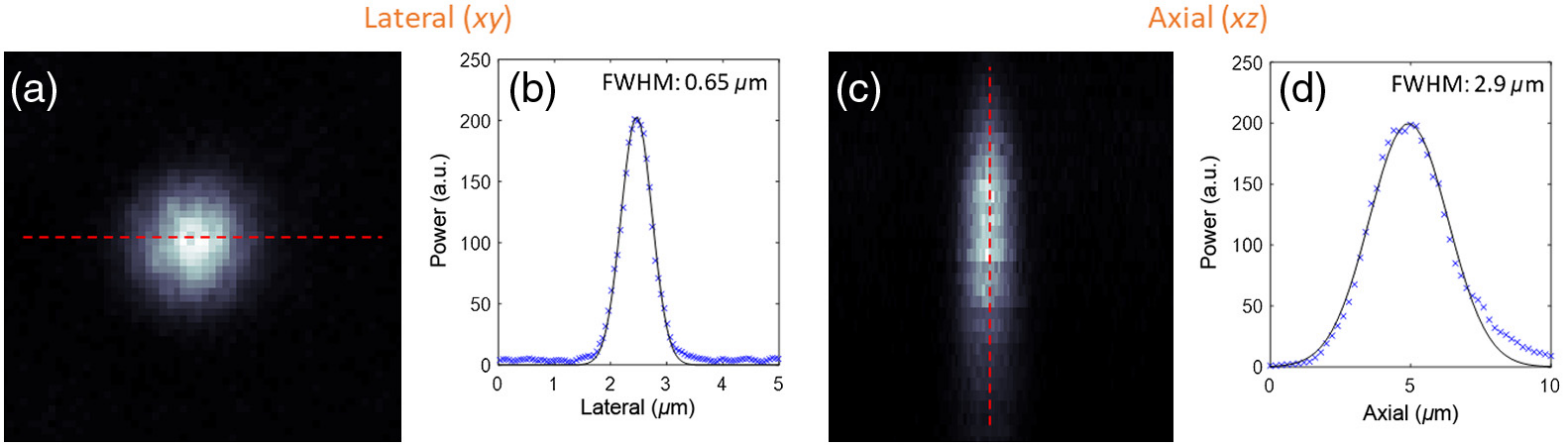
PSF measurements. (a) The lateral FWHM is 0.65  μm. (b) The axial FWHM is 2.9  μm.

The multiphoton microscope is shown in the red shaded region of Fig. 1. The system was designed de novo around the aforementioned 1300-nm fiber laser, for cost efficiency and performance optimization. Galvo mirrors (GVS002, Thorlabs, Newton, New Jersey, United States) are used to scan the beam. The scan and tube lenses comprise of several air-spaced commercial lenses from Thorlabs. The optical design was optimized in Zemax to minimize aberrations, resulting in diffraction-limited performance for optical ray angles below 11.3 deg. The scan lens consists of two LA1541-C lenses and a LA1031-C lens and the tube lens consists of two AC508-300-C. The signals are epi-collected by a 20× (NA=1) objective lens (XLUMPLFLN20XW, Olympus, Tokyo, Japan), separated by a 775-nm longpass dichroic mirror (FF775-Di01, Semrock Inc., Rochester, New York, United States), and then detected sequentially using emission filters by a photomultiplier tubes (PMT) (H7422-40, Hamamatsu Photonics, Hamamatsu, Japan). The collection path was designed in Zemax from commercial lenses (Thorlabs) to ensure maximum collection efficiency. The first lens (Thorlabs LA1256-A) is followed by an air-spaced lens consisting of three plano-convex lenses (Thorlabs LA1417-A). This optical design ensures a full collection field-of-view (FOV) of ∼1  mm in diameter. The sample stage enabled coarse lateral movement and axial scanning using a three-dimensional (3D) stepper motor (TRA25CC, Newport Corp., Irvine, California, United States). A moveable silver mirror downstream of the dichroic mirror enables optional widefield imaging. The pre-amp is a FEMTO DHPCA-100 (FEMTO Messtechnik GmbH, Berlin, Germany), and the data acquisition card is an NI PCIe-6353 (National Instruments Corp., Austin, Texas, United States). Samples were excited using the same wavelength and pulse duration, but images were sequentially collected beginning with the signal requiring the least pulse energy (i.e., SHG) to minimize the risk of photodamage. The power was adjusted using a combination of neutral density filters, and polarization control was employed to maximize the signal strength. Images were collected with specific bandpass-filters to isolate SHG, THG, and 3PE fluorescence signals. Through the appropriate choice of filters crosstalk between images was avoided, as evident from the signals being delocalized between the images. The power dependency of the three signals was verified by collecting emission as a function of illumination beam power. The microscope system was controlled using ScanImage and the images were processed using ImageJ software (Fiji Distribution, Version 1.52e).^23^^,^^24^ Frame averaging was used to further minimize noise. Colors were arbitrarily assigned to the grayscale images. The pulse duration was measured using an autocorrelator after the objective lens at 114 fs. The point spread function (PSF) of the system was measured using 175-nm blue fluorescent beads; the results for the lateral (0.65  μm) and axial (2.9  μm) directions are shown in Fig. 3. For calibration and PSF measurements, the fiber laser was operated at a repetition rate of 1 MHz. For biological sample imaging, the repetition rate of the laser was increased to 10 MHz to improve scanning efficiency. In both instances, the maximum pulse energy at the sample plane was ∼10  nJ.

A summary of the optical performance of the MPM system is given in Table 1.

**Table 1:** Performance of MPM system.

Specification	Value
Wavelength	∼1300 nm
Maximum pulse energy (post objective)	10 nJ
Pulse duration (post objective)	114 fs
Maximum peak power (post objective)	87 kW
Maximum average power (post objective)	100 mW
Repetition rate	10 MHz
MPM system loss	6.6 dB
Lateral PSF (FWHM)	0.65 μm
Axial PSF (FWHM)	2.9 μm
FOV	1 × 1 mm
Spatial mode	Gaussian

### 2.4 Biological Samples

Experimental protocols were approved by the Mass Eye and Ear Internal Review Boards, and all methods were carried out in accordance with relevant guidelines and regulations. Fresh frozen sections of healthy human motor branch of obturator nerve and transgenic mice sciatic nerve were employed. Adult human nerve samples (patient age >18 years) were obtained fresh at the time of the free-tissue transfer for smile reanimation after informed consent among patients undergoing facial reanimation procedures. Sciatic nerves from thy1-YFP mice were harvested in accordance with Massachusetts Eye and Ear Institutional Animal Care and Use Committee approval. Specifically, animals were placed in an induction chamber and placed under general anesthesia by inhalation of 2% isoflurane in 0.6  l/min $O_{2}$. Once unconscious, isoflurane was increased to 5% and continued until cessation of breathing was noted for one minute, followed by immediate cervical dislocation. Nerves were immediately harvested from the carcasses. Whole mounts were kept at 4°C in saline-soaked gauze and imaged fresh within 2 h. Nerves for sectioning were processed using a previously described protocol.^25^ Briefly, nerves were fixed by immersion in 2% phosphate-buffered paraformaldehyde, followed by cryoprotection in sucrose solution, cryosectioning at 1 to 2  μm, and staining using a non-toxic myelin-specific dye followed by mounting on glass slides prior to multiphoton imaging.

## 3 Experimental Results

System performance and laser parameters including PSF, pulse energy, and duty cycle were optimized to enhance image contrast and acquisition time while minimizing photodamage. The use of 1300 nm permits simultaneous multiphoton excitation and multiharmonic generation microscopy while avoiding undesirable two-photon absorption from endogenous fluorophores (namely, NADH and FAD), with subsequent cross-talk and photodamage.

### 3.1 Imaging of Human Obturator Nerve

**Fig. 4:**
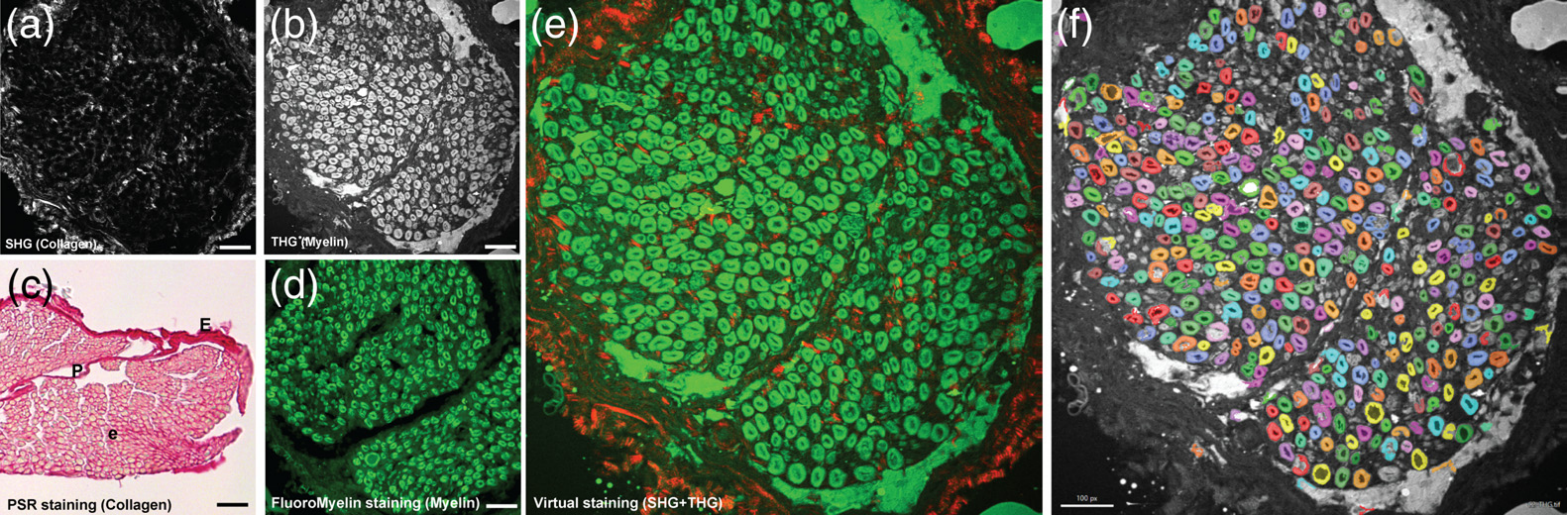
Label-free imaging of *ex vivo* human obturator nerve cross-sections using a novel 1300-nm fiber laser-powered multiphoton microscope. (a) Second-harmonic generation imaging of neural connective tissue collagen, post objective average power 7.5 mW. (b) Third-harmonic generation imaging of myelinated fibers, post objective average power 15 mW. (c) Picrosirius red staining of myelinated fibers (conventional light microscopy). (d) Myelin-specific dye (FluoroMyelin Green, Molecular Probes, Eugene, Oregon, United States) staining of cross-section (widefield fluorescence microscopy). (e) Merged image combining SHG (red) and THG (green) signals. (f) Automated histomorphometry of peripheral nerve human obturator nerve section imaged by THG. The FOV is 380×380  μm, and the scale bar is 50  μm.

The microscope was used for SHG and THG epi-detection imaging of human and murine peripheral nerve. Figures 4(a) and 4(b) show images of axially frozen-sectioned human obturator nerve obtained sequentially with different bandpass filters (BPF). For this label-free sample, the SHG signal (617/73 nm BPF) was generated from collagen in the three nerve sheath layers (endoneurium, perineurium, and epineurium). Collagen is the main tissue component responsible for an SHG signal in peripheral nerve tissue. For validation of label-free SHG imaging, picrosirius red was employed on separate tissue sections as an efficient alternative to immunohistochemistry for high-specificity labeling of collagen-dense structures. Picrosirus red-stained collagen was imaged using brightfield microscopy^26^ as shown in Fig. 4(c). A THG signal (420/50-nm BPF) was predominately generated from the myelin sheaths of myelinated axons [Fig. 4(b)], demonstrating near-identical imaging results compared to widefield fluorescence imaging of myelin-specific dye stained sections, as shown in Fig. 4(d). Though the THG signal is predominantly emitted in the forward direction, there is sufficient backward-scattering in turbid media (such as peripheral nerve) to obtain a sufficient signal using epi-detection as demonstrated here and by other groups.^9^^,^^27^ The combination of photobleaching-free SHG and THG images reveal complementary information allowing characterization of nerve morphology in unstained tissues, as shown in Fig. 4(e). Figure 4(f) shows myelinated axon quantification of unlabeled cryosectioned human peripheral nerve performed through segmentation of a THG image using commercial machine learning software.^28^

### 3.2 Imaging of Sciatic Murine Nerve

**Fig. 5:**
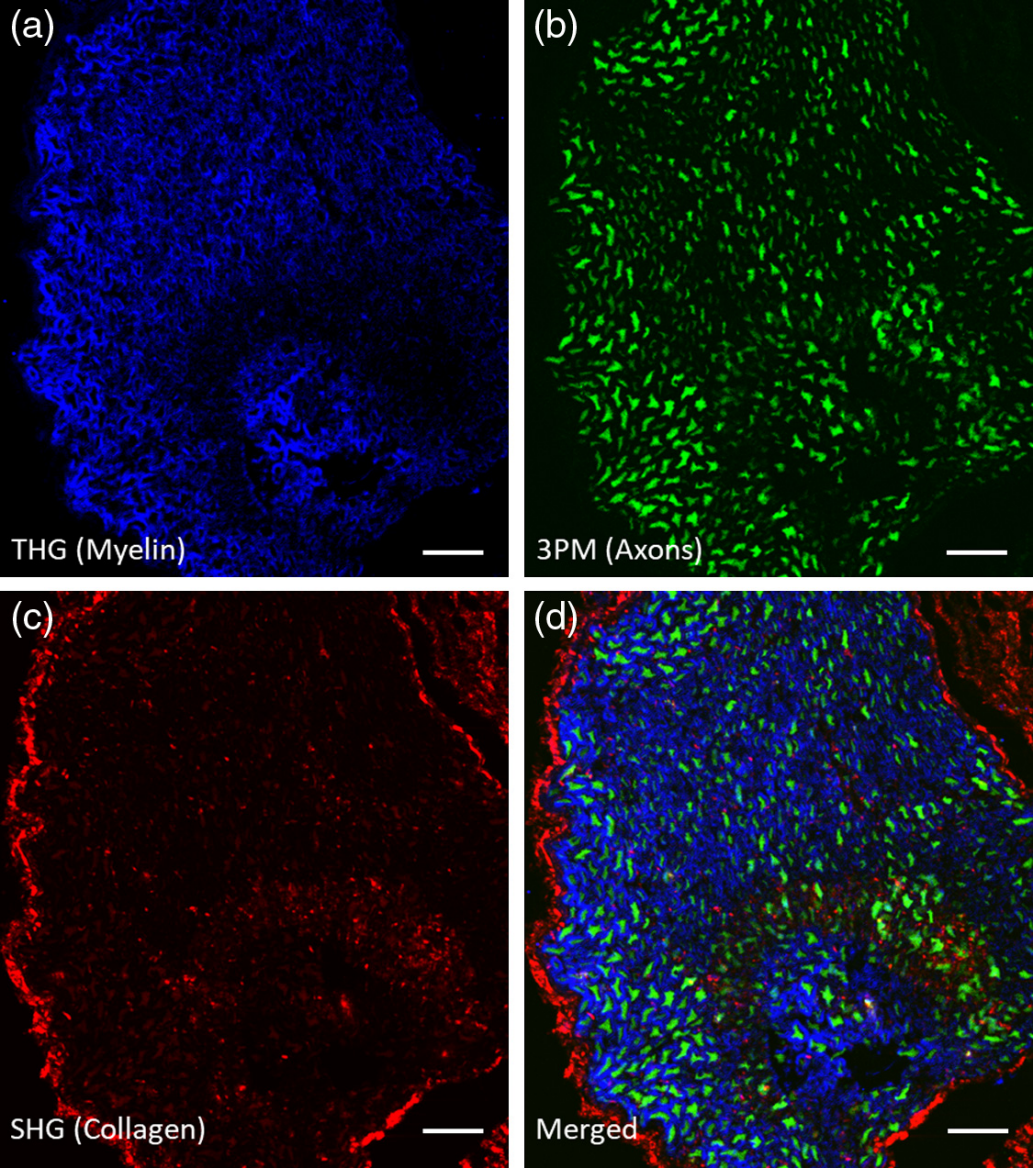
Multiphoton-microscopy image of *ex vivo* sciatic nerve of a thy1-YFP mouse. FOV is 210×240  μm. Scale bars are 25  μm. Post objective average power was 12.5 mW. (a) Third-harmonic generation (blue) shows the myelinated fibers. (b) Three-photon excited fluorescence of YFP (green) shows axons. (c) Second-harmonic generation (red) shows collagen fibers. (d) Merged image of THG, 3PE, and SHG signals.

Figure 5 shows imaging of a sciatic nerve cross-section from a thy1-YFP mouse using the multiphoton microscope developed here. Three-photon-excitation fluorescence images were collected using a third filter (535/60  nm BPF) to separate them from SHG and THG signals. Figures 5(a)–5(c) show a THG signal from myelin, SHG signal from collagen, and three-photon fluorescence excitation signal from YFP-labeled axons. In Fig. 5(d), the three images are merged to obtain a multicolor image.

**Fig. 6:**
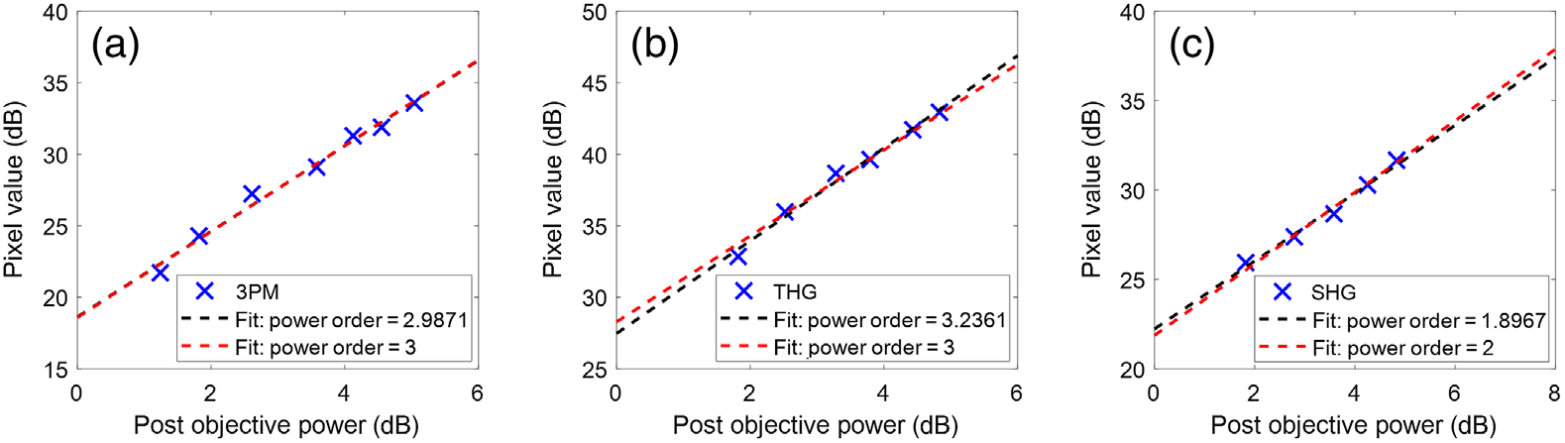
Power dependence of the three signals measured in Fig. 5. (a) Three-photon signal. (b) THG signal. (c) SHG signal.

To estimate the nonlinear order and required power for multiphoton imaging, the dependence of the signal on excitation power was measured. The plot of signal strength versus excitation power is shown in Fig. 6, along with fits. The black-dashed lines represent the power order as a free-fitting parameter, and the red-dashed lines represent fits with fixed power order. The fits show excellent agreement with the data, indicating capture of appropriate emission. Excitation power exhibits a second-order increase in the SHG signal (×1.90) and third-order increase in the 3PE (×2.99) and THG (×3.24) signals.

**Fig. 7:**
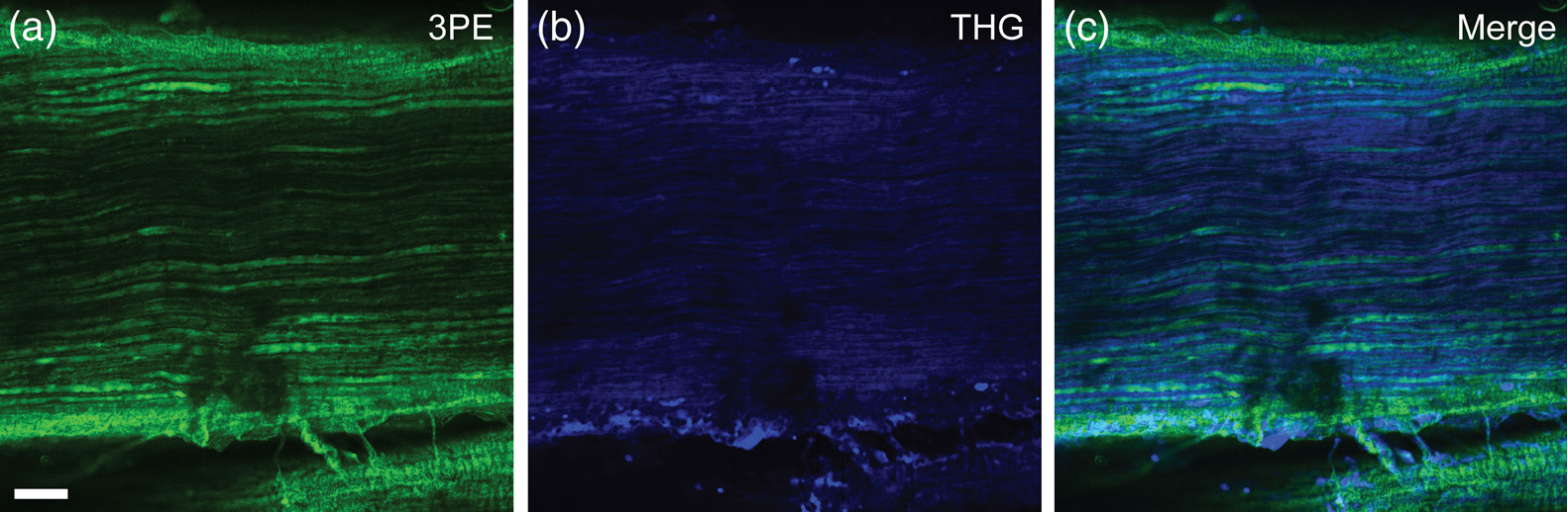
Multiphoton-microscopy images of wet mounted sciatic nerve of a thy1-YFP mouse at a depth of 120  μm, post objective average power was 30 mW. (a) Three-photon excited fluorescence of YFP (green) shows axons. (b) Third-harmonic generation (blue) signal shows the myelinated fibers. (c) Merged image of 3PE and THG signals. About 50 frame averages were used to enhance image quality. FOV is 480×480  μm. Scale bars is 50  μm.

To assess whether this novel 1300-nm fiber-based multiphoton microscope could potentially be employed for *in vivo* imaging of mammalian nerve, freshly harvested whole mount murine sciatic nerve from a thy1-YFP mouse was imaged. Figure 7 shows the results for 3PE [Fig. 7(a), YFP] and THG imaging [Fig 7(b), myelin] using non-descanned epi-detection at a depth of 120  μm into the tissue. The two images are merged in Fig. 7(c).

## 4 Discussion

Disorders of myelination are cardinal features of several inherited and acquired diseases of the nervous system.^17^ Noninvasive neuroimaging techniques based on magnetic resonance imaging are useful for diagnosis and monitoring of several demyelinating diseases,^29^ but lack resolution necessary for quantitation of individual fibers. In surgery for peripheral nerve dysfunction, means for rapid and reliable quantification of axons present within nerve branches could inform operative decision making.^30^^–^^33^ The present study demonstrated the potential of label-free multiphoton microscopy paired with rapid cryosection techniques for quantitation of myelinated axons in human nerve relevant to clinical practice. In peripheral nerves of transgenic reporter mice, stain-free multicolor imaging epineurial sheaths, unmyelinated and myelinated axons were achieved using a single excitation wavelength, demonstrating the utility of this microscopy platform as a research tool.

Nerve histomorphometry based on conventional axial sectioning approaches necessitates nerve biopsy, yielding loss of neural function. A key advantage of THG microscopy over other high-resolution imaging techniques is its potential for resolution of individual myelinated fibers within intact peripheral nerve, as has been previously demonstrated using an 1180-nm laser illumination source.^9^ Though THG imaging of central nervous system tissue has been achieved at depths up to a few hundred microns, imaging of peripheral axons in intact peripheral nerve is challenging owing to the highly scattering properties of the epineurial sheath. Heretofore, two-photon excitation imaging of myelinated axons within intact mammalian peripheral nerve has been limited depths <70  μm.^34^ Here, we achieved an imaging depth exceeding 100  μm within intact excised peripheral nerve using 3PE at 1300 nm. In comparison to illumination below 1200 nm, longer illumination wavelengths for THG imaging yields longer wavelength emission in the visible (>400  nm) as opposed to ultraviolet (<400  nm) domains, lessening undesirable signal absorption to permit deeper imaging. Longer illumination wavelengths also increase achievable imaging depth of SHG of collagen.^35^

Several commercial femtosecond lasers exist with outputs at 1300 nm. However, these lasers may lack the necessary peak power to achieve THG. As shown in the Supplementary Materials, additional sections were imaged using a commercial multiphoton microscope. Despite identical PMT and BPF and similar epi-collection non-descanned optical paths, insufficient signal was captured to resolve THG and 3PE signals (Fig. S1 in the Supplementary Materials).

Future work will examine means to optimize lateral and axial resolution in multiphoton microscopy. An epi-detection NLOM imaging signal is typically collected via non-descanned detection, using a large area detector positioned close to the objective lens to minimize signal loss along the collection path. Alternatively, descanned confocal detection may potentially be employed to minimize background signal and increase axial resolution, though loss of signal along the descanned detection path and pinhole may negate potential gains in the signal-to-background ratio. In place of a pinhole and single point detector, an array of photodetectors (e.g., Zeiss Airyscan detector, Carl Zeiss AG, Jena Germany) paired with deconvolution algorithms could be alternatively employed to optimize a descanned detection signal by capturing photons that would otherwise be discarded by the pinhole.^36^ Spatial resolution could be further enhanced by digital pixel reassignment or blind image reconstruction, as previously reported for two-photon emission microscopy.^37^^,^^38^

## 5 Conclusion

We developed a novel microscope based on a custom-built 1300-nm excitation source for multi-harmonic and multi-photon excitation microscopy. We demonstrated the utility of the microscope in label-free imaging of intact peripheral nerves and frozen sections of healthy murine sciatic nerve and healthy human obturator nerve. Three-photon fluorescence, SHG, and THG imaging were achieved using a cost-efficient custom fiber-based high-powered near-infrared femtosecond laser source. Despite a long illumination wavelength at 1300 nm, an adequate SHG signal to highlight collagen-rich endoneurium, perineurium, and epineurium was obtained via non-descanned epi-detection. The combination of SHG and THG imaging using a novel single fiber femtosecond laser source at 1300 nm represents means for label-free imaging of peripheral nerve with potential clinical implications for non-ablative neural histomorphometry.

## Code, Data, and Materials Availability

Code and data can be made available upon request to the corresponding author.
