# Supplementary material for: Multiphoton microscopy for label-free multicolor imaging of peripheral nerve
Source: J Biomed Opt. 2022 May 16;27(5):056501. doi: 10.1117/1.JBO.27.5.056501 (PMC9109936; doi:10.1117/1.JBO.27.5.056501)
Supplement: Supplementary file 1 [file JBO_027_056501_SD001.pdf]

Supplementary Information for:

Multiphoton microscopy for label-free multicolor imaging of peripheral nerve

Lars Rishøj,[a,c] Iván Coto Hernández,[b] Siddharth Ramachandran,[a] and Nate Jowett[b]
[a]Boston University, ECE, Boston, USA
[b]Mass Eye and Ear and Harvard Medical School, Surgical Photonics and Engineering Laboratory, Boston, USA
[c]Currently: Technical University of Denmark, DTU Fotonik, Kgs. Lyngby, Denmark

1. Supplementary information

Supplemental Fig. 1 demonstrates the lack of THG and 3PE signal when imaging an axial section of Thy1-YFP facial nerve stained with a myelin specific dye (FluoroMyelin Red®, F34652 Invitrogen, Carlsbad, CA) using a commercial multiphoton microscope (TrimScope II, LaVision BioTech GmbH) powered by a commercial femtosecond laser (Spectra-Physics Insight X3, Newport Corp, Newport, NJ) at 1300 nm owing to insufficient peak power. Note strong 2PE fluorescence signal in panel A from excitation of YFP and FMR at 920 nm.

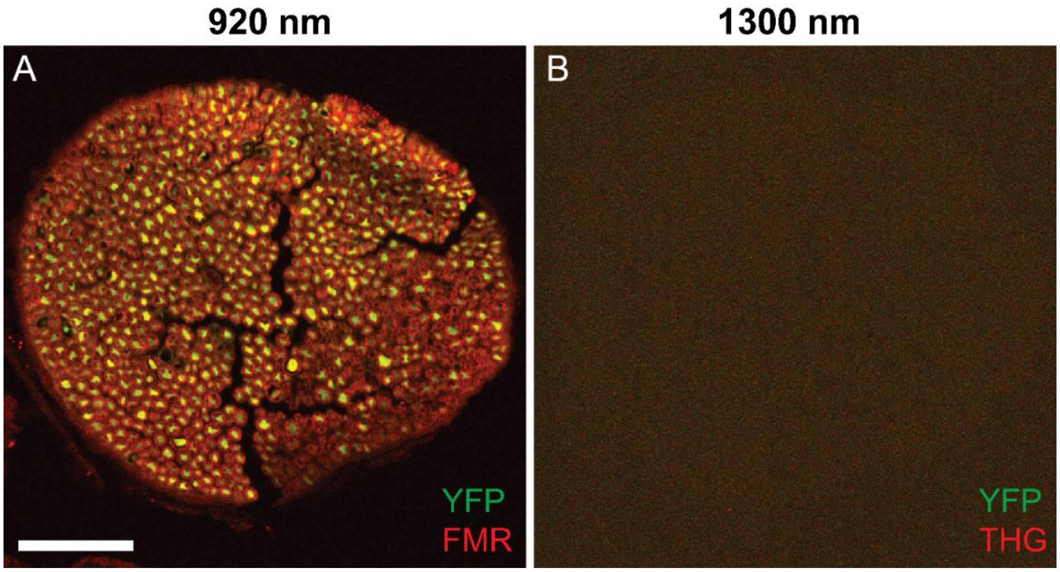

Suppl. Fig. 1. Cross-section of the buccal branch of the facial nerve in Thy1-YFP-16 mouse stained with a myelin-specific dye imaged using a commercial multiphoton microscope powered by a commercial dual-output femtosecond laser. (**A**) Two-photon fluorescence microscopy image (excitation 920 nm) demonstrates axon (YFP) and myelin signal (FMR). (**B**) Same volume imaged at maximal output power at 1300 nm demonstrated absent THG and 3PE signals. Scale bar 100 μm.
